# A new protocol for a single-stage combined cardiopulmonary and echocardiography exercise test: a pilot study

**DOI:** 10.1093/ehjimp/qyae021

**Published:** 2024-03-21

**Authors:** Dan M Dorobantu, Curtis A Wadey, Ben Berryman, Nurul H Amir, Lynsey Forsythe, A Graham Stuart, Guido E Pieles, Craig A Williams

**Affiliations:** Children’s Health and Exercise Research Centre (CHERC), Public Health and Sports Sciences, Faculty of Life and Health Sciences, University of Exeter Medical School, St Luke’s Campus, Heavitree Road, Exeter EX1 2LU, UK; Population and Translational Health Sciences, University of Bristol, Bristol, Beacon House, Queens Rd, BS8 1QU, UK; Children’s Health and Exercise Research Centre (CHERC), Public Health and Sports Sciences, Faculty of Life and Health Sciences, University of Exeter Medical School, St Luke’s Campus, Heavitree Road, Exeter EX1 2LU, UK; Children’s Health and Exercise Research Centre (CHERC), Public Health and Sports Sciences, Faculty of Life and Health Sciences, University of Exeter Medical School, St Luke’s Campus, Heavitree Road, Exeter EX1 2LU, UK; Population and Translational Health Sciences, University of Bristol, Bristol, Beacon House, Queens Rd, BS8 1QU, UK; Faculty of Sport Science and Recreation, Universiti Teknologi MARA, Perlis Branch, Arau Campus, Uitm Arau, 02600 Arau, Malaysia; Congenital Heart Unit, Bristol Royal Hospital for Children and Heart Institute, Upper Maudlin St, BS2 8BJ, Bristol, UK; Congenital Heart Unit, Bristol Royal Hospital for Children and Heart Institute, Upper Maudlin St, BS2 8BJ, Bristol, UK; Sports Cardiology and Screening Department, ASPETAR Qatar Orthopaedic and Sports Medicine Hospital, Sports City Street, Inside Aspire Zone, Al Buwairda St, Doha, Qatar; Institute of Sport Exercise and Health, University College London, 170 Tottenham Ct Rd, W1T 7HA, London, UK; Children’s Health and Exercise Research Centre (CHERC), Public Health and Sports Sciences, Faculty of Life and Health Sciences, University of Exeter Medical School, St Luke’s Campus, Heavitree Road, Exeter EX1 2LU, UK

**Keywords:** stress test, multimodal cardiac evaluation, heart failure, cardiac function, advanced cardiac imaging

## Abstract

**Aims:**

The value of cardiopulmonary exercise testing (CPET) and exercise stress echocardiography (ESE) in managing cardiac disease is well known, but no standard CPET–ESE protocol is currently recommended. This pilot study aims to compare feasibility and cardiac function responses between a new high-intensity single-stage combined test (CPET–hiESE) and a standard maximal ESE (smESE).

**Methods and results:**

After screening and maximal CPET, all volunteers (*n* = 21) underwent three ESE modalities: (i) based on the gas exchange threshold (hiESE–GET, 40% of peak-GET, 6 min), (ii) based on heart rate (HR) (hiESE–HR, 80% of peak HR, 6 min), and (iii) smESE (85% of predicted peak HR for age, 3 min). Speckle tracking echocardiography (STE) and tissue Doppler imaging (TDI) were measured at each step. There was superior image quality and data completeness for the right ventricle strain for both hiESE modalities compared with smESE (71.4 and 76.2 vs. 42.9%, *P* = 0.07). Left ventricular STE data completeness was similar for all three conditions. Despite systematically higher HR, work rate and levels of exertion in the smESE compared with hiESE, STE and TDI parameters were not systematically different. Concordance correlation coefficients ranged from 0.56 to 0.88, lowest for strain rate parameters and mean difference from −0.34 to 1.53, highest for TDI measurements.

**Conclusion:**

The novel CPET–hiESE protocol allowed for better data completeness, at lower levels of exertion compared with smESE, without systematically different cardiac reserve measurements in healthy participants. This single-stage protocol can be individualized to clinical populations, which would provide practical advantages to standard testing.

## Graphical Abstract

**Graphical abstract:**
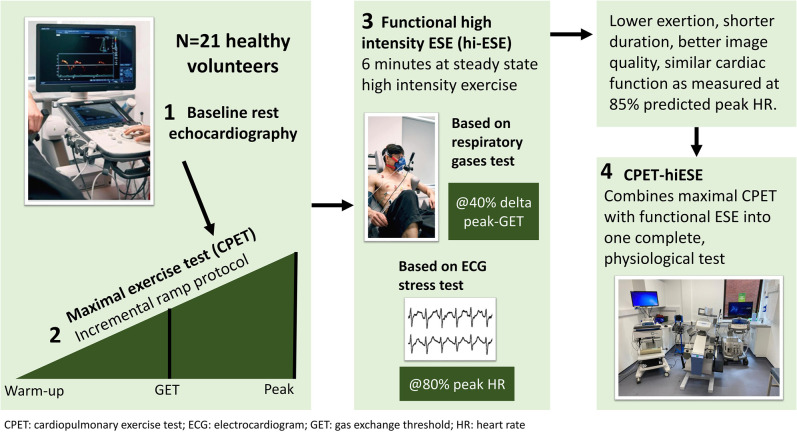
Proposed combined cardiopulmonary exercise test—high-intensity exercise stress echocardiography (CPET–hiESE) protocol.

## Introduction

Exercise intolerance, dyspnoea, or fatigue are common complaints associated with cardiovascular disease, making exercise testing one of the most useful clinical tools available.^1,2^ Cardiopulmonary exercise testing (CPET) is the gold standard for assessing exercise peak performance and a decrease in parameters such as oxygen uptake (VO_2_) is associated with cardiovascular disease prognosis.^1^ Nevertheless, CPET cannot always indicate the pathophysiological causes of symptoms or worsening of exercise tolerance, due to the complex oxygen transport and utilization pathways.^1,2^

Exercise stress echocardiography (ESE) was originally introduced to evaluate myocardial viability and ischaemia, in coronary heart disease.^3^ In the past decade, assessment of cardiac functional exercise reserve gained applications in a wide spectrum of clinical scenarios.^4^ CPET–ESE has thus been implemented in clinical practice and research, as it can provide a comprehensive evaluation of cardiorespiratory function.^2,5–8^ It combines the advantages of both CPET and ESE but has no current standard methodology, which negatively impacts adoption and uniform practice.^2^ In addition, important limitations of current protocols have emerged when used in conjunction with state-of-the-art imaging.^5^

Adapting CPET protocols for ESE is a complex task, as both modalities will have different needs and technical limitations. Current guidelines recommend that CPET is performed using an incremental ramp protocol, with comprehensive maximal testing criteria.^9^ These guidelines move away from such maximal test criteria as predicted peak heart rate (HR) values, respiratory exchange ratio thresholds, or rate of perceived exertion (RPE) used in isolation.^9^ On the other hand, cardiac imaging guidelines recommend using a step testing protocol such as modified Bruce or McMasters,^3,4^ which are further modified within centres, resulting in considerable variability in practice.^2^ These incremental step testing protocols have numerous limitations when combined with state-of-the-art imaging, owing to short acquisition intervals, movement/respiratory artefacts, and poor definitions for exercise intensity domains, and especially what constitutes ‘peak’ effort.^2,4^ In effect, despite having the potential of being ‘true’ quantitative tests, ESE guidelines still provide semi-qualitative thresholds for ‘abnormal’ results, usually based on single studies, which is not optimal, especially for longitudinal follow-up.^4^

Many of the above limitations could be addressed by the integration of CPET (or maximal ECG testing) into individualizing ESE protocols. The relationship between stroke volume, VO_2_, work rate, and HR is complex and non-linear throughout the exercise, with various responses from the plateau to bimodal or progressive increase,^10,11^ influenced by age, fitness, pathology.^10^ Additionally, it appears that HR is the main driver of cardiac output increase at high-intensity exercise in the supine position rather than stroke volume, making submaximal ESE a potentially valuable tool.^11,12^ Although there have been some studies attempting such submaximal methods, these are often within a moderate rather than a high-intensity exercise domain.^7,13–15^ Our previous work with conventional CPET–ESE highlighted that left ventricular function measured at an intensity above moderate intensity, more strongly correlated to peak VO_2_ in healthy athletes, making this a more suitable choice.^16^

Therefore, we propose a steady-state high-intensity ESE (hiESE) CPET protocol (CPET–hiESE) for the comprehensive evaluation of cardiorespiratory peak performance and exercise functional reserve. This combines a maximal CPET to determine respiratory parameters and submaximal thresholds (e.g. the gas exchange threshold [GET]) followed by a single-stage 6 min hiESE to evaluate biventricular cardiac functional exercise reserve. The submaximal intensity threshold was set to allow for heavy exercise for longer periods of time, which is not possible at peak exertion.^17^ This pilot study with healthy volunteers, aims to compare the feasibility and cardiac reserve parameters between two hiESE modalities (40% of the difference between peak and GET—hiESE–GET, and 80% of observed peak HR—hiESE–HR) and a standard step test ‘peak’ step (85% of predicted HR for age, standard maximal ESE [smESE]). We hypothesized that the CPET–hiESE protocol would provide non-inferior feasibility for speckle tracking echocardiography (STE) and tissue Doppler imaging (TDI) analysis, lower work rates, HR, and perceived exertion, without systematically lower values of cardiac function parameters, when compared with smESE. The proposed protocol would potentially improve reproducibility and use of complex exercise testing in cardiac disease, with benefits in streamlining the workflow, and integration of more complex modalities such as cardiac magnetic resonance or three-dimensional echocardiography.

## Methods

### Participants

This study was advertised among University of Exeter staff between February and April 2023, with an approximate target of 20 participants (10 under 35 years old and 10 above 35 years). The study was approved by the University of Exeter Faculty of Health and Life Sciences, Sport and Health Sciences Ethics Committee (reference number 626294). The inclusion criteria were no known relevant acute of chronic health conditions, defined as cardiac or pulmonary conditions, or those that would affect exercise performance or introduce risks, assessed on a case-by-case basis by two study team clinicians. Exclusion criteria were absolute or relative contraindication to exercise^9^; recent (less than 6 months) surgery/catheter intervention; pregnancy as declared by participant; and current or ex-smoker.

After informed consent was obtained, volunteers underwent screening by general physical examination, electrocardiogram, and resting echocardiography. A total of 26 volunteers underwent screening, of which 3 were referred to further investigations for suspected cardiac conditions. One participant stopped the maximal test early due to a leg discomfort test and was excluded. One participant had very poor views on rest and exercise and was excluded from post-processing. This results in a total of 21 volunteers being included in the analysis.

### Study protocol

All volunteers underwent a health questionnaire, including a self-reported assessment of habitual physical activity (sedentary or low activity, casual or regular leisure activity, and highly trained or athlete). Stature, body mass, age, body composition (InnerScan Body Composition Monitor, Tanita Corporation, Tokyo, Japan), resting HR, ECG (Cardio 100BT, Custo Med Gmbh, Ottobrunn, Germany), and blood pressure were documented. A resting supine echocardiogram was obtained, following the current guidelines.^18^

### Maximal CPET

CPET was performed on a semi-recumbent (45° inclination) cycle ergometer (eBike II EL, GE Medical Systems, Milwaukee, Wisconsin, USA). After a warm-up of unloaded pedalling (3 min), the work rate increased at a rate of 15–30 W min^−1^, based on sex, stature, and physical activity level. Breath-by-breath measurements were done using a calibratet metabolic cart (Metamax 3B, CORTEX Biophysik GmbH, Leipzig, Germany). Participants were fitted with oronasal masks and breathing valve assemblies (V-mask 7400 series, Hans Rudolph, Shawnee, USA). Subjective exercise intensity (10-point Borg scale) and blood pressure were measured every two minutes, as well as continuous ECG throughout the exercise and recovery (Cardio 100 BT, Custo Medical, Ottobrunn, Germany). Test was ended when at least 2 of the following were met: exertion or leg fatigue was graded at 9–10 on the Borg scale, a pedalling frequency of 60 rpm could not be maintained despite active encouragement, or a VO_2_ plateau despite work rate increase.^9^

### High-intensity exercise threshold calculations

Gas exchange measurements were exported in 10 s average (Metasoft Studio v5.9.2, CORTEX Biophysik GmbH, Leipzig, Germany) in standard tabular format (csv/xls). The GET was identified using the V-slope method,^19^ defined as the point on the VCO_2_/VO_2_ plot at which the VCO_2_ starts to increase more rapidly than VO_2_ and was cross-checked using the ventilation equivalent for O_2_ method.^20^ The following thresholds were used for the experimental conditions: (i) high (heavy) intensity derived from gas exchange data, set at the work rate corresponding to 40% delta (Δ; i.e. 40% of the difference between the VO_2_ at GET and peak exercise) (hiESE–GET), chosen to allow for a comfortable exercise duration of 6 min, with homogeneous physiological responses;^17,21,22^ (ii) high intensity derived from measured peak HR, set at the work rate corresponding to an HR of 80% from the peak (hiESE–HR), which is within the high-intensity domain, below the threshold for severe intensity exercise and should approximate the 40% delta threshold;^23^ and (iii) current practice acceptable maximal test step as defined by current stress imaging guidelines, set at the work rate corresponding to an HR of 85% of predicted maximal HR for age (220-age) (smESE). The 40% Δ intensity and 6-min duration were chosen based on what was previously used in a cohort of cystic fibrosis patients (at a higher intensity of 60% Δ),^22^ to ensure that this is not perceived as too difficult by participants, especially when translated to future clinical populations with impaired exercise capacity. Previous work by Lansley *et al*. did show 40% Δ provided the optimal perceived exertion levels for prolonged exercise, which was the aim here.^17^ The work rate at 40% delta was calculated using a linear regression between work rate and VO_2_ during the ramp test, adjusting using the ramp rate for a mean response time of 30 s.^17,24^

### Exercise echocardiography experimental protocols

The order of the three experimental conditions was randomized. The hiESE–GET and hiESE–HR bouts were set at 6 min and the smESE bout was set at 3 min, with an immediate transition from a 2-min warm-up at 20 W. Continuous ECG was monitored for safety. Concomitant gas exchange was not measured, as it was not the aim of the study.

Echocardiography was performed using an Aplio i900 machine (Canon Medical Systems, Japan), with the semi-recumbent cycle ergometer at a 45° inclination, and left tilting within participant comfort. Imaging was performed at baseline in a standard supine position, 10 min after the maximal CPET on the bicycle (to assess image quality) and then during exercise, starting 1 min into each experimental condition, to allow for transition from Phase I (cardio-dynamic) kinetics and stabilization of stroke volume and HR and images were acquired until the end of each bout.^25^ A rest period of 10 min was given before each exercise bout. The following 2D views (8 continuous seconds per view) were obtained: 2D apical left ventricle (LV) 4 chamber (LV-A4C), 2 chamber (LV-A2C), 3 chamber (LV-A3C), right ventricle (RV) focused, and short axis LV (SAX mitral and papillary muscle level). LV ejection fraction (LVEF) and RV fractional area change (RV-FAC) as secondary outcomes were measured from standard apical views (see Supplementary data online). Pulsed wave tissue Doppler (TDI) at the base of the tricuspid annulus, mitral septal and mitral lateral levels were acquired. Peak systolic (Sʹ) and diastolic (Eʹ) velocities were measured (RV-Sʹ, RV-Eʹ, septal LV-Sʹ, septal LV-Eʹ, lateral LV-Sʹ, and septal LV-Eʹ). When Eʹ and Aʹ fusion was noted during exercise, the wave was considered Eʹ. During scanning, after acquiring all needed views once, additional 8-s loops were taken for views that initially appeared suboptimal. Acquiring SAX views was difficult, as previously described in other studies,^15^ with 45% of participants having low quality or difficult SAX views on the semi-recumbent position, more common in women due to different types of sportswear impeding the views. As such, we only report the feasibility for SAX views analysis, agreement analysis not being possible due to few data point pairs (*n* = 7).

### Speckle tracking echocardiography (STE)

All described 2D views were acquired at 80–120 frames per second and analysed offline (Ultra Extend NX; Canon Medical Systems, Japan), per current guidelines.^26^ The endocardial border was traced (automatically preferred, manually otherwise), and myocardial thickness was adjusted. Automated six segment tracking was used on one selected best cardiac cycle, using both LV and RV dedicated packages. Tracking quality and strain curves were visually inspected, and tracking was adjusted as necessary. If more than two segments (one for RV free wall tracking) were not appropriately tracked, the view was excluded. The following parameters were obtained from STE: peak longitudinal systolic strain (S_l_) and peak longitudinal systolic strain rate (SR_l_). These are obtained for each segment of the myocardium and averaged: LV global longitudinal (12 segment maximum and 4 segments minimum) and RV free wall (RVFW) longitudinal (3 segments maximum and 2 segments minimum).

### Statistical analyses

Frequencies are given as numbers and percentages, with continuous values as mean ± standard deviation (SD). Achieved data completeness was compared between groups using the Fisher’s test.

Mean values for HR, work rate, and cardiac function parameters for each condition were compared, including standard supine imaging as a baseline. To account for repeated measures and data missing at random within each condition, linear mixed models with participant cluster as the random effect term (intercept) and the Kenward–Roger approximation of degrees of freedom were used to calculate *P*-values, to limit Type-1 error inflation when analysing. The random effects models were compared with the linear model using the likelihood ratio test, and a significant random effect was observed for all models (*P* < 0.05).

To assess agreement between the test method pairs, three methods were used: (i) Bland–Altman plots with percentage outside of limits of agreement (LoA) as a measure of outlier frequency, 95% LoA, as a measure of variability and mean difference as a measure of systematically fixed bias^27^; (ii) Lin’s concordance coefficient, as a measure of both precision and accuracy to determine how far the observed data deviate from the line of perfect concordance (higher coefficient represents better linear correlation)^27^; and (iii) Pitman–Morgan correlation between difference and mean with Bradley–Blackwood omnibus test of equality of means and variances, to assess whether there is proportional bias, affecting values with higher means more, with the Bradley–Blackwood *P* < 0.05 and positive Pitman–Morgan coefficient denoting lower concordance at higher values.^28,29^

Inter- and intra-observer reliability were assessed with Lin’s concordance coefficient and Bland–Altman mean difference, on exercise strain measurements, randomly selecting 10 RV, A4C, A3C, and A2C, equally distributed between smESE, hiESE–HR, and hiESE–GET. Reliability for stress TDI was reported previously in the literature.^30^

## Results

A total of 21 volunteers completed the protocol, ranging from 23 to 60 years old (described in *Table 1*). On average, the feasibility of STE analysis was better in hiESE–GET and hiESE–HR compared with smESE for LV-A2C, LV-A3C, RV, and short axis views (*Table 2*) but not LV-A4C. This resulted in higher (data not statistically significant) feasibility in both hiESE–GET and hiESE–HR compared with smESE for RV STE (71.4 and 76.2 vs. 42.9%, *P* = 0.07) and slightly better feasibility for RV-S′ (100 and 100 vs. 90.5%, *P* = 0.1).

**Table 1:** Participant general characteristics

Age, years	41.5 ± 11.4 (23–60)
Gender, *n*	
Female	8 (38.1)
Male	13 (61.9)
Body mass, kg	74.7 ± 10.8 (56.2–91.7)
Stature, cm	174 ± 9 (160–189)
BSA, cm^2^	1.89 ± 0.15 (1.57–2.15)
BFP, *n* (%)	24.5 ± 8.9 (11.1–44.7)
Rest systolic BP, mmHg	132 ± 9 (111–143)
Rest diastolic BP, mmHg	78 ± 11 (59–101)
Rest HR, bpm	58 ± 10 (47–84)
Regular physical activity, *n* (%)^a^	
Casual	14 (66.7)
Highly trained	7 (33.3)

BFP, body fat percentage; BP, blood pressure; BSA, body surface area; HR, heart rate.

^a^Physical activity is self-assessed during the health questionnaire. Among the highly trained, two participants were amateur endurance athletes.

**Table 2:** Proportion of obtainable ventricular function measurements, by modality and protocol type

*n* (%)	Rest (on bicycle) (*n* = 19)^a^	hiESE–GET (*n* = 21)	hiESE–HR (*n* = 21)	smESE (*n* = 21)	*P* value^b^
STE					
LV-A4C	19 (100)	20 (95.2)	20 (95.2)	21 (100)	0.6
LV-A2C	18 (94.7)	16 (76.2)	17 (81)	13 (61.9)	0.5
LV-A3C	18 (94.7)	15 (71.4)	14 (66.7)	14 (66.7)	0.9
RV	18 (94.7)	15 (71.4)	16 (76.2)	9 (42.9)	0.07
LV-SAXb^c^	9 (81.8)	7 (63.6)	4 (36.4)	5 (45.5)	0.6
LV-SAXm^c^	11 (100)	7 (63.6)	4 (36.4)	5 (45.5)	0.6
Pulsed wave TDI					
LV lateral	19 (100)	21 (100)	21 (100)	21 (100)	n/a
LV septal	19 (100)	21 (100)	21 (100)	21 (100)	n/a
RV lateral	19 (100)	21 (100)	21 (100)	18 (85.7)	0.3

A2C, apical two-chamber view; A3C, apical three-chamber view; A4C, apical four-chamber view; hiESE-GET, high-intensity exercise stress echocardiography based on gas exchange threshold; hiESE-HR, high-intensity exercise stress echocardiography based on peak heart rate; LV, left ventricle; RV, right ventricle; SAX, short axis view; SAXb, basal SAX view; SAXm, mid/papillary muscle SAX view; smESE, standard maximal exercise stress echocardiography; STE, speckle tracking echocardiography; TDI, tissue Doppler imaging.

^a^In two participants rest views were not acquired on the semi-recumbent cycle.

^b^*P* value from in-between group comparison for the three experimental modalities, excluding rest.

^c^SAX views at rest on the semi-recumbent cycle were of good quality for *n* = 11, and feasibility during exercise was calculated based on these.

**Figure 1:**
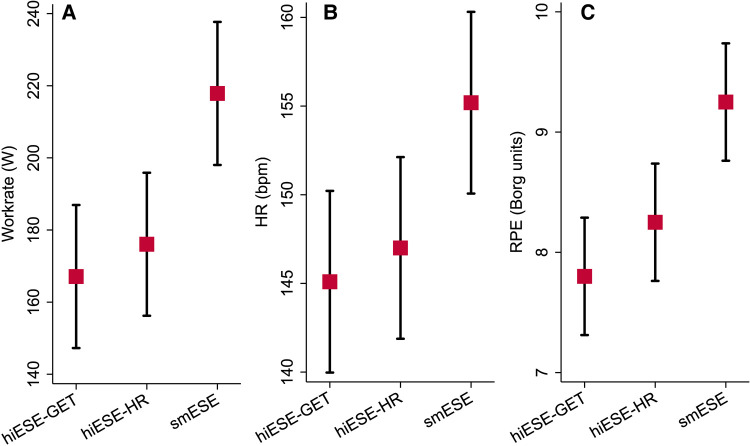
Exercise testing parameters, by protocol type. (*A*) Exercise work rate, (*B*) exercise heart rate (HR), and (*C*) end-exercise rate of perceived exertion (RPE). Red squares (black-capped vertical lines) represent marginal estimates (95% confidence interval) from repeated measures linear mixed models with test type as fixed effect and participant identity as random effect terms. Mean ± standard deviation values shown in *Table 3*.

**Figure 2:**
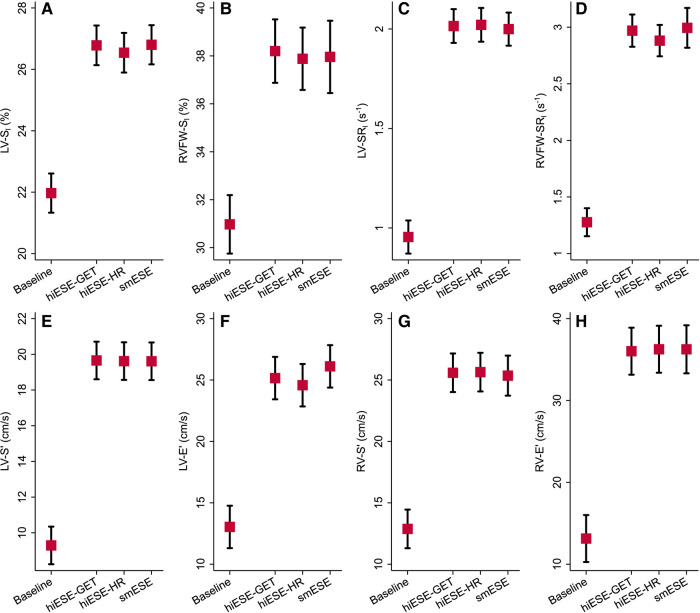
Speckle tracking echocardiography (STE) and tissue Doppler imaging (TDI)-derived cardiac function parameters, by protocol type, in relation to baseline. (*A*) Global left ventricular (LV) longitudinal strain (LV-S_l_), (*B*) right ventricle (RV) free wall longitudinal strain (RVFW-S_l_), (*C*) LV longitudinal strain rate (LV-SR_l_), (*D*) RV free wall longitudinal strain rate (RVFW-SR_l_), (*E*) average left ventricular (LV) peak systolic mitral annulus velocity (LV-S′), (*F*) average LV peak diastolic mitral annulus velocity (LV-E′), (*G*) right ventricle (RV) peak systolic tricuspid annulus velocity (RV-S′), and (*H*) RV peak diastolic tricuspid annulus velocity (RV-E′). Red squares (black-capped vertical lines) represent marginal estimates (95% confidence interval) from repeated measures linear mixed models with test type as fixed effect and participant identity as random effect terms. Mean ± standard deviation values shown in *Table 3*.

HR, work rate, and RPE were higher for smESE compared with both hiESE–GET and hiESE–HR (*Figure 1*), but conventional, STE, and TDI cardiac function parameters were similar across the three modalities (*Figure 2* and *Table 3*). Data on LVEF and RV–FAC agreement between modalities is presented in Supplementary data online, *Table S1* and *Figure S1*. There was no evidence of systematically higher values for smESE compared with hiESE–GET and hiESE–HR, but there was low or moderate paired measurement agreement. STE and TDI data are detailed below.

**Table 3:** Conventional STE and TDI cardiac function at baseline and during exercise during smESE, hiESE–GET, and hiESE–HR

	Baseline (rest)	hiESE–GET	hiESE–HR	smESE
LVEF, %	61.7 ± 5.1	70.5 ± 3.9	70.9 ± 4.1	71.1 ± 5.9
RV-FAC, %	44.7 ± 4.9	58.5 ± 6.1	59.9 ± 6.2	59.8 ± 4.6
STE				
LV-S_l_, %	22 ± 1.7	26.8 ± 1.6	26.6 ± 1.4	26.8 ± 1.1
RV-S_l_, %	31 ± 2.9	38.5 ± 3.3	37.9 ± 2.4	39.2 ± 2.5
LV-SR_l_, s^−1^	0.96 ± 0.94	2.01 ± 0.25	2.01 ± 0.21	2 ± 0.2
RV-SR_l_, s^−1^	1.17 ± 0.21	2.98 ± 0.35	2.85 ± 0.32	3.04 ± 0.3
TDI				
LV-Sʹ, cm/s	9.3 ± 1.5	19.6 ± 2.8	19.6 ± 2.8	19.6 ± 2.5
LV-Eʹ, cm/s	13 ± 2.4	25.2 ± 4.7	24.6 ± 4.4	26.1 ± 4.2
RV-Sʹ, cm/s	12.9 ± 1.9	25.6 ± 4.6	25.6 ± 3.8	25.3 ± 3.9
RV-Eʹ, cm/s	13.1 ± 2.9	36 ± 7.1	26.3 ± 8.3	36.4 ± 7.5
Testing parameters				
HR, bpm	58 ± 10	145 ± 14	147 ± 11	155 ± 10
Work rate, W	0	167 ± 39	176 ± 42	218 ± 56
RPE, Borg units	0.1 ± 0.5	7.8 ± 1.2	8.3 ± 1.2	9.3 ± 0.9

E′, peak diastolic annular velocity; FAC, fractional area change; hiESE–GET, high-intensity exercise stress echocardiography based on gas exchange threshold; hiESE–HR, high-intensity exercise stress echocardiography based on peak heart rate; HR, heart rate; LoA, limits of agreement; LV, left ventricle; LVEF, LV ejection fraction; RPE, rate of perceived exertion; RV, right ventricle; S′, peak systolic annular velocity; smESE, standard maximal exercise stress echocardiography; S_l_, peak systolic longitudinal strain; SR_l_, peak systolic longitudinal strain rate.

Values are means ± standard deviation (SD). Baseline (rest) data are from a standard supine echocardiography.

### Agreement between smESE and hiESE–GET testing

**Figure 3:**
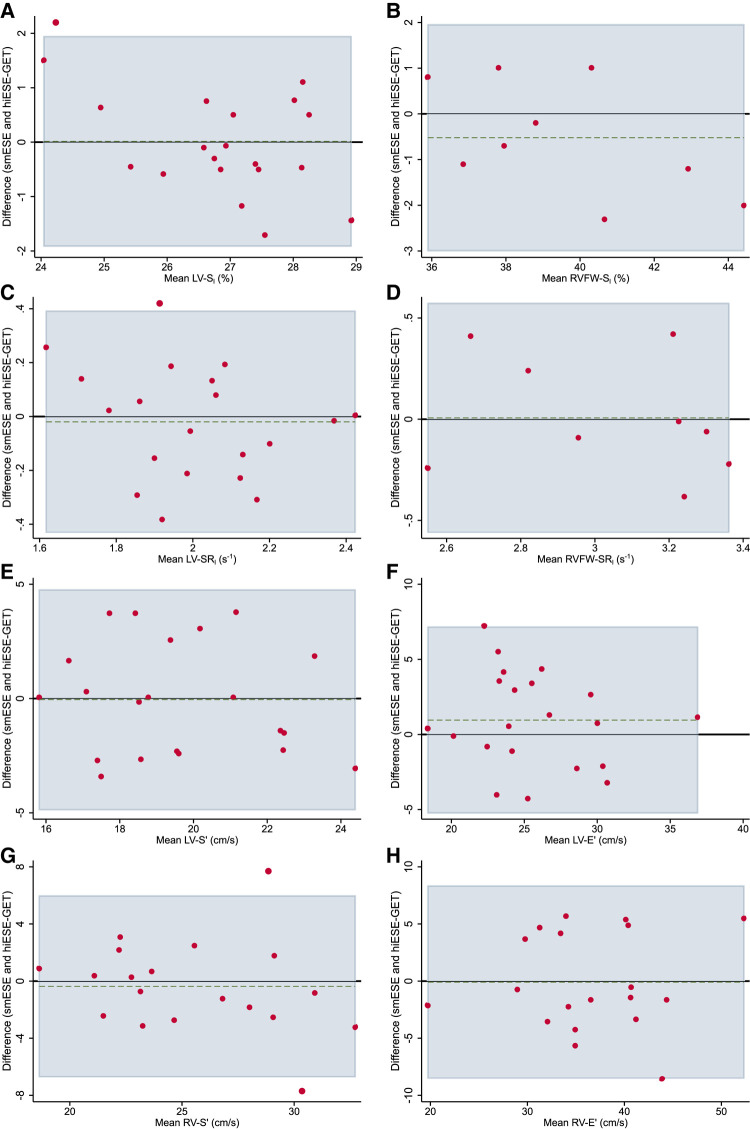
Bland–Altman plots showing agreement between smESE and hiESE–GET testing for cardiac function parameters. (*A*) Global left ventricular (LV) longitudinal strain (LV-S_l_), (*B*) right ventricle (RV) free wall longitudinal strain (RVFW-S_l_), (*C*) LV longitudinal strain rate (LV-SR_l_), (*D*) RV free wall longitudinal strain rate (RVFW-SR_l_), (*E*) average LV peak systolic mitral annulus velocity (LV-S′), (*F*) average LV peak diastolic mitral annulus velocity (LV-E′), (*G*) RV peak systolic tricuspid annulus velocity (RV-S′), and (*H*) RV peak diastolic tricuspid annulus velocity (RV-E′). Black horizontal line shows null mean difference. Horizontal green dashed line shows actual mean difference. Light blue area shows 95% limits of agreement (LoA). Red dots show data pairs.

There was no evidence of systematically higher cardiac function measurements in smESE compared with hiESE-GET (*Figure 3*, *Tables 3* and *4*), despite systematically higher HR (mean difference 10 bpm, *P* < 0.001), work rate (mean difference 51 W, *P* < 0.001), and RPE (mean difference 1.5 Borg units, *P* < 0.001). There was a good agreement between testing conditions (*Table 4*), with a wider LoA and a higher proportion of values outside of the LoA in TDI-derived parameters, and lower concordance coefficient in SR_l_ parameters. There was no evidence of significant proportional bias identified by the Bradley–Blackwood test.

**Table 4:** Agreement between smESE, hiESE–GET, and hiESE–HR testing modalities

	LoA 95% CI	Mean difference	Outside LoA, *n* (%)	Concordance correlation	Difference–mean correlation	Bradley–Blackwood *P* value
smESE vs. hiESE–GET						
LV-S_l_	[−1.9; 1.9]	0.01	1 (5)	0.76	−0.51	0.07
RV-S_l_	[−3; 1.9]	−0.52	0 (0)	0.88	−0.57	0.14
LV-SR_l_	[−0.43; 0.39]	−0.02	1 (5)	0.57	0.25	0.52
RV-SR_l_	[−0.56; 0.57]	0.01	0 (0)	0.62	−0.25	0.79
LV-Sʹ	[−4.8; 4.8]	−0.05	0 (0)	0.58	−0.14	0.82
LV-Eʹ	[−5.2; 7.1]	0.96	1 (4.8)	0.73	−0.18	0.29
RV-Sʹ	[−6.6; 5.9]	−0.34	2 (10.5)	0.71	−0.24	0.54
RV-Eʹ	[−8.5; 8.3]	−0.06	1 (5.3)	0.83	0.05	0.98
smESE vs. hiESE–HR						
LV-S_l_	[−1.3; 1.8]	0.24	1 (5)	0.79	−0.43	0.07
RV-S_l_	[−4.6; 3.9]	−0.31	0 (0)	0.53	−0.24	0.8
LV-SR_l_	[−0.43; 0.36]	−0.03	0 (0)	0.46	−0.13	0.67
RV-SR_l_	[−0.41; 0.51]	0.05	0 (0)	0.54	0.31	0.67
LV-Sʹ	[−4.5; 4.5]	−0.01	1 (4.8)	0.63	−0.15	0.8
LV-Eʹ	[4.5; 7.5]	1.53	0 (0)	0.7	−0.07	0.1
RV-Sʹ	[−4.9; 4.6]	−0.17	1 (5.3)	0.81	−0.04	0.94
RV-Eʹ	[−8.7; 9]	0.18	0 (0)	0.84	−0.26	0.54
hiESE–GET vs. hiESE–HR						
LV-S_l_	[−1.2; 1.5]	0.14	1 (5.3)	0.9	0.37	0.19
RV-S_l_	[−2.3; 2.3]	0.01	1 (7.7)	0.9	0.28	0.64
LV-SR_l_	[−0.38; 0.35]	−0.02	0 (0)	0.67	0.17	0.73
RV-SR_l_	[−0.47; 0.53]	0.03	0 (0)	0.7	0.1	0.87
LV-Sʹ	[−4.3; 4.3]	0.04	1 (4.8)	0.69	0	0.99
LV-Eʹ	[−5.8; 7]	0.58	2 (9.5)	0.74	0.11	0.66
RV-Sʹ	[−4.9; 4.8]	−0.05	2 (9.5)	0.83	0.32	0.37
RV-Eʹ	[−7.1; 6.6]	−0.24	1 (4.8)	0.9	−0.34	0.29

CI, 95% confidence interval; E′, peak diastolic annular velocity; hiESE–GET, high-intensity exercise stress echocardiography based on gas exchange threshold; hiESE–HR, high-intensity exercise stress echocardiography based on peak heart rate; LoA, limits of agreement; LV, left ventricle; RV, right ventricle; S′, peak systolic annular velocity; smESE, standard maximal exercise stress echocardiography; S_l_, peak systolic longitudinal strain; SR_l_, peak systolic longitudinal strain rate.

### Agreement between smESE and hiESE–HR testing

**Figure 4:**
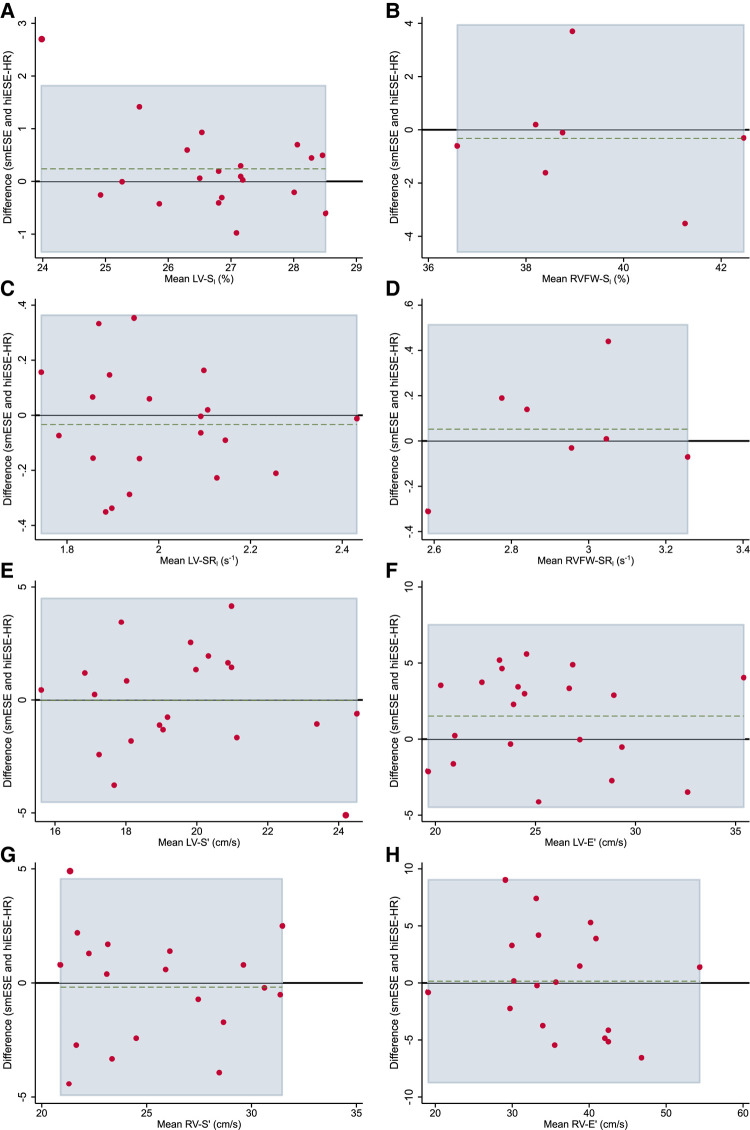
Bland–Altman plots showing agreement between smESE and hiESE–HR testing for cardiac function parameters. (*A*) Global left ventricular (LV) longitudinal strain (LV-S_l_), (*B*) right ventricle (RV) free wall longitudinal strain (RVFW-S_l_), (*C*) LV longitudinal strain rate (LV-SR_l_), (*D*) RV free wall longitudinal strain rate (RVFW-SR_l_), (*E*) average LV peak systolic mitral annulus velocity (LV-S′), (*F*) average LV peak diastolic mitral annulus velocity (LV-E′), (*G*) RV peak systolic tricuspid annulus velocity (RV-S′), and (*H*) RV peak diastolic tricuspid annulus velocity (RV-E′). Black horizontal line shows null mean difference. Horizontal green dashed line shows actual mean difference. Light blue area shows 95% limits of agreement (LoA). Red dots show data pairs.

There was no evidence of systematically higher cardiac function measurements in the smESE compared with hiESE–HR testing (*Figure 4*, *Table 4*), despite systematically higher HR (mean difference 8 bpm, *P* < 0.001), work rate (mean difference 42 W, *P* < 0.001), and RPE (mean difference 1 Borg units, *P* < 0.001). *Table 4* details good agreement between testing conditions, but overall worse for SR_l_ and RV parameters, with wide LoA for TDI-derived measurements. There was no evidence of significant proportional bias identified by the Bradley–Blackwood test.

### Agreement between hiESE–GET and hiESE–HR testing

**Figure 5:**
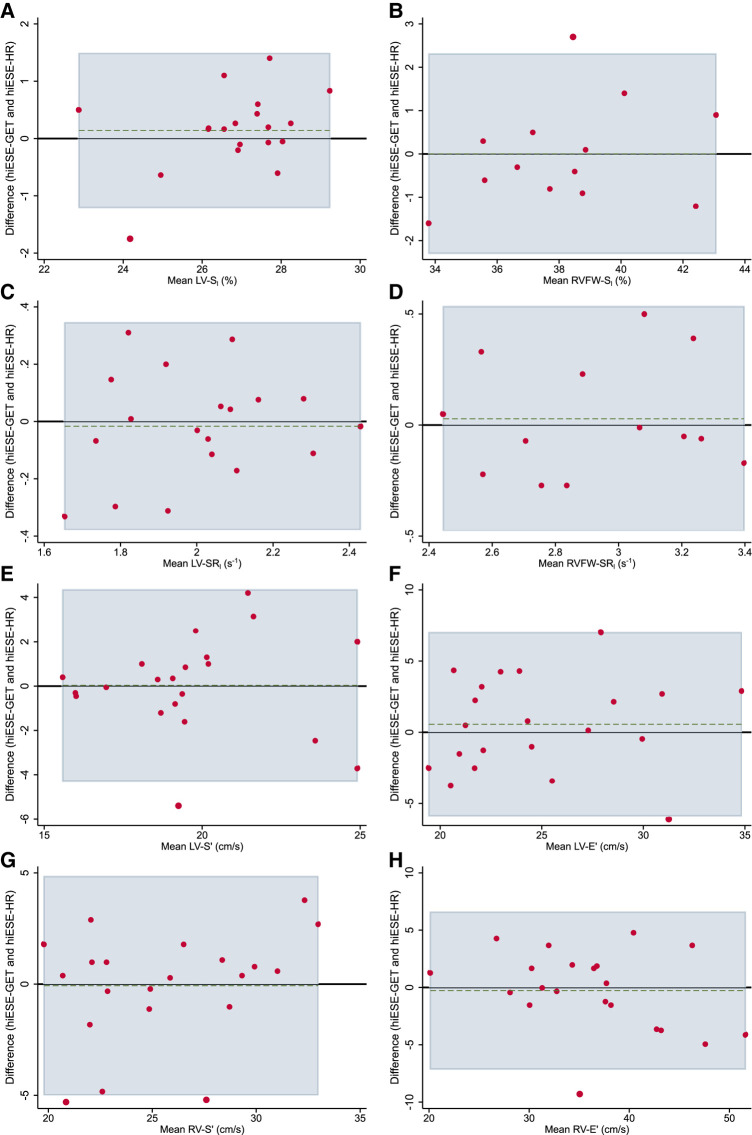
Bland–Altman plots showing agreement between hiESE–GET and hiESE–HR testing for cardiac function parameters. (*A*) Global left ventricular (LV) longitudinal strain (LV-S_l_), (*B*) right ventricle (RV) free wall longitudinal strain (RVFW-S_l_), (*C*) LV longitudinal strain rate (LV-SR_l_), (*D*) RV free wall longitudinal strain rate (RVFW-SR_l_), (*E*) average LV peak systolic mitral annulus velocity (LV-S′), (*F*) average LV peak diastolic mitral annulus velocity (LV-E′), (*G*) RV peak systolic tricuspid annulus velocity (RV-S′), and (*H*) RV peak diastolic tricuspid annulus velocity (RV-E′). Black horizontal line shows null mean difference. Horizontal green dashed line shows actual mean difference. Light blue area shows 95% limits of agreement (LoA). Red dots show data pairs.

There was a very good agreement in cardiac function measurements between the two testing conditions, with wider LoA for TDI-derived parameters (*Figure 5*, *Table 4*). In addition, the HR was similar (mean difference 2 bpm, [min −21; max 15], *P* = 0.4), with a concordance coefficient of 0.78. Between the two, hiESE–HR testing had a higher work rate (mean difference 9 W, [min −44; max 26], *P* = 0.08, concordance coefficient 0.88), and RPE (mean difference 0.5 Borg units, *P* = 0.08), detailed in *Table 4*.

### Reliability for hiCPET–ESE STE

For both the RV and LV, inter- and intra-observer reliability was very good or excellent, and LV strain reliability was similar between smESE, hiESE-GET, and hiESE–HR (*Table 5*).

**Table 5:** Inter- and intra-observer reliability for high-intensity exercise stress STE

	Interobserver		Intraobserver	
	Concordance correlation	Mean difference	Concordance correlation	Mean difference
LV-S_l_ (A4C)	0.81	−0.12 ± 0.8	0.82	−0.37 ± 0.6
LV-S_l_ (A3C)	0.68	−0.66 ± 0.8	0.79	−0.1 ± 0.6
LV-S_l_ (A2C)	0.89	−0.14 ± 0.7	0.82	−0.29 ± 0.9
RVFW-S_l_	0.86	−1.31 ± 1.4	0.91	−0.68 ± 1.5
smESE all LV-S_l_	0.75	−0.16 ± 0.8	0.92	−0.21 ± 0.5
hiESE-GET all LV-S_l_	0.81	−0.25 ± 0.8	0.71	−0.36 ± 0.9
hiESE-HR all LV-S_l_	0.82	−0.59 ± 0.6	0.86	−0.18 ± 0.6

A2C, apical two-chamber view; A3C, apical three-chamber view; A4C, apical four-chamber view; hiESE–GET, high-intensity exercise stress echocardiography based on gas exchange threshold; hiESE–HR, high-intensity exercise stress echocardiography based on peak heart rate; HR, heart rate; LV, left ventricle; RV, right ventricle; smESE, standard maximal exercise stress echocardiography; S_l_, peak systolic longitudinal strain; STE, speckle tracking echocardiography.

Concordance correlation is Lin’s concordance coefficient. The mean difference is expressed as mean ± standard deviation from the Bland–Altman method. RV was not reported within each experimental condition due to too few measurements.

## Discussion

This is the first proposed combined CPET–ESE protocol that uses information obtained from a maximal CPET (or ECG test if CPET is not available) to streamline and standardize a subsequent hiESE, thus addressing many of the limitations that are inherent to conventional incremental step test protocols. Advanced ESE is being used more in practice and has the potential to be a very sensitive and comprehensive prognostic tool, as recently showcased in a large multicentre focused on pulmonary hypertension^8^ or previously in heart failure.^2^ This pilot study showed that a steady-state hiESE, combined with a CPET (CPET–hiESE; Graphical Abstract) is a technically feasible alternative to a standard maximal step test ESE (smESE) in healthy volunteers, a first step towards clinical use. It also had better quality and more efficient image acquisition, at a lower perceived exertion and work rate, and with less time spent imaging and offline processing due to fewer steps compared with standard ESE protocols. The core aim of the proposed new protocol is for a single-stage ESE to be combined with maximal CPET and concomitant gas exchange measurements, in one coherent framework, with results that can be interpreted as a whole, even if done sequentially. This also results in streamlined acquisition and analysis, with just one ESE step, adding also peak performance data from the CPET, to detailed mechanistic data from ESE.

As hypothesized, we did not observe systematically different biventricular cardiac function parameters in neither of the three modalities in healthy individuals, as beyond high-intensity exercise, the main driver in cardiac output increase is expected to be HR rather than stroke volume, especially in semi-supine exercise.^11,12^ This further adds to the potential value of submaximal ESE, as a reproducible and feasible alternative to currently available ‘peak’ effort-based protocols, especially in conjunction with modern imaging techniques, such as STE. Because of the individualized nature of the thresholds, based on measured, rather than estimated performance, this protocol can be flexible, while maintaining the personalized nature. This should allow for future modifications to fit special clinical population, such as ESE at moderate-intensity exercise, based on GET, longer recovery pauses, concomitant ESE–CPET measurements or accelerated ramp protocols.

### Rationale for submaximal ESE in clinical practice

During incremental exercise, cardiac output does not have a linear relationship with VO_2_ (and subsequently HR and work rate), the rate of cardiac output change decreases beyond high-intensity effort.^10,11^ The relationship of stroke volume (SV) with VO_2_ (and HR or work rate) is even more complex, with a steep increase up to and including moderate-intensity exercise, and then a slow gradual increase, a plateau, or a decrease, up to peak, with how and why these variable patterns occur being likely related to the fitness levels, clinical situation, and the relative contribution of HR to the total cardiac output.^11,12^

We have previously shown that in adolescent athletes and healthy children, cardiac function increases only modestly beyond moderate-intensity exercise.^5,16^ There have been previous studies using variations of steady-state submaximal exercise, but all were within the moderate-intensity domain.^7,13–15,31^ Previous studies, especially when using STE, either limit the peak exercise to when imaging is still feasible^30^ or impose an HR upper limit,^15^ to allow for a good balance between purpose and usability. In a cohort of healthy athletes, LV-S_l_ measured at high, and not moderate intensity exercise, correlated to peak VO_2_.^16^ Due to the non-linear nature of SV changes with exercise intensity^11^ and describes tendency of biventricular strain to plateau at high intensity,^31,32^ it is reasonable to hypothesize that any increases between high intensity and severe exercise domains would be even lower in relative magnitude than those seen between moderate and high intensities, making the latter domain a very good candidate for submaximal ESE.

In the current pilot study, we compared cardiac function parameters at submaximal, high-intensity physiological thresholds, with one based on 85% of predicted peak HR, a step clinicians would consider a test valid and maximal in practice.^4^ A previously proposed ESE protocol in children and adolescents sets a submaximal imaging step at 75–85% of predicted peak HR for age,^33^ resulting in work rates and exercise HR quite similar to those found in our smESE modality, and describes an LV-S_l_ reserve of 17%, comparable to what we observed across the three modalities tested here. Often, using predicted peak HR equations does not reflect the population being tested, since those are based on small samples, or general populations with fewer elderly or underrepresentation of ethnicities, physical activity levels, etc.^34^ Even with significantly higher work rates, HR and perceived exertion, cardiac function parameters were not systematically higher in smESE compared with hiESE, which is explained by the above-discussed non-linear relation of SV and cardiac output with exercise intensity.^10^

A key feature of this study is the provision of experimental data to support the potential of individualized submaximal hiESE in clinical practice. This method could be sensitive enough to capture abnormal cardiac reserve measurements, while avoiding difficult, unreproducible, and unreliable peak exertion evaluations. By using an individualized high-intensity threshold, based either on gas exchanges or peak HR data, cardiac function imaging should be performed at similar levels of exertion in patients with a wide range of fitness levels. Importantly, basing high-intensity threshold calculations on true, measured and individual metabolic data should allow for more accurate calculations of work rate that allow for testing. This should permit hiESE even in populations with exercise limitations, as these calculations will follow peak performance, even when reduced. In fact, a study using a similar, GET-peak delta concept, set at a higher 60% Δ, showed the feasibility of a 6-min bout even in a population with severe lung disease, namely, cystic fibrosis.^22^ Equally important, moderate-intensity testing can also be implemented using the same framework, for example, at 90% of GET work rate, a modification to the protocol which is currently being implemented in our centre.

### Combined CPET–ESE—a matter of true integration

The currently used incremental step ESE protocols, the most common modifications of the original Bruce protocol, dating back to the 1960s, have several technical and conceptual limitations when applied to modern quantification of cardiovascular function, which create barriers in adoption and standardization. These include short imaging windows, arbitrary intensity domains, poor definitions of peak exercise, difficult imaging at high levels of exertion, and lack of common physiological denominator among very different populations.

When establishing a combined CPET–ESE protocol, we needed to consider that a maximal cardiorespiratory fitness test and a cardiac functional reserve test are not meant for the same purposes, do not answer the same clinical questions, and as such, should not expect that a similar methodology would fit both. Our team, leading up to the development of the currently reported pilot study, had been using an incremental step test CPET–ESE, with a modified Bruce protocol, with concomitant metabolic and echocardiographic measurements at each step.^5,16^ Most currently used CPET–ESE protocols are similar, with CPET being performed simultaneously, or at a separate time. Current guidelines, also reflected in a recent review, provide some recommendations for which of the steps are of critical importance, and an attempt to anchor these in exercise intensity domains is being made.^4,14,33^ The aim should be for more standardized methods to be implemented for CPET–ESE.

To address many of these limitations of functional exercise testing, we propose integrating a maximal CPET (or standard ECG exercise test when CPET is not available) into the way ESE is delivered, to allow for individual testing parameters for each patient, improved testing reliability and better use of state-of-the-art imaging techniques. The data from this pilot study are encouraging, in which we have shown good feasibility of the method, and that it compared favourably to already existing, standard, protocols, without systematic measurement bias. STE and TDI showed very good absolute agreement between testing conditions, while RV-FAC and LVEF had a low or moderate agreement, which follows what has been shown previously on rest imaging.^35–37^ We speculate that the semi-automated measurements, high frame rates, and the drift correction contributed to this for STE. High feasibility during scanning and high-temporal frame rate likely contributed to good reliability for TDI. Conventional manually measured LVEF and RV-FAC have neither of these advantages, and it might be that test–retest reliability during exercise is affected more by less-than-optimal endocardial border definition and movement artefact than it is in STE, where built-in algorithms account for this.

Importantly, concomitant gas exchange measurements during the ESE 6-min steps should be implemented in clinical practice, even if this was not done here in order to simplify the protocol. This can provide data on the relationships between cardiac function, cardiac output, and metabolic parameters, shown previously to be of clinical importance.^2,38,39^ The aim of this study was to show ESE reliability and feasibility and gas exchange data were not required. A larger healthy volunteer cohort is planned, as well as pilot clinical studies in relevant populations, to establish the role of CPET–hiESE in routine and research practice, which will include two exercise intensities and concomitant ESE and gas exchange measurements.

### Limitations

This is a pilot study in healthy individuals only, biased towards physically active individuals, and requires further testing in other populations to assess generalizability and clinical role. Due to this being a pilot study, the sample size was small, as the aim was to evaluate methodological feasibility. The quality of the SAX views was suboptimal in many of the participants and as such this analysis was limited. This study was designed based on the variability of STE and TDI in our lab, and as such other parameters which are less reliable during exercise, like LVEF and RV-FAC were not included to ensure statistical power. Also, the focus of the pilot study was on STE and TDI, with LVEF and RV-FAC being secondary parameters, with potentially higher variability in measurements, for which the sample size was not adequately large. As such these secondary outcomes results presented in the Supplementary data online should be interpreted with caution, as the low reliability observed could be a result of inadequate statistical power. An intermediate step at moderate intensity was not included to simplify the protocol and shorten the visit time for the volunteers, but future studies plan to include this, currently underway at our centre. It was not the aim of the study to evaluate the use of other maximal testing modalities (such as an upright bicycle) in conjunction with ESE. STE is frame rate dependent, and during exercise low frame rate could lead to underestimation of peak strain values. In this current study, image optimization and the use of modern equipment led to high frame rates of over 80 fps, limiting the impact of this source of error. Lastly, the rest period before each test was short, at 10 min, which might not allow for full return to baseline, but with random-order counterbalancing, we believe the bias was minimal, and for future clinical studies, it can be extended as needed.

## Conclusion

This healthy volunteer pilot study showed proof-of-concept for integrating a maximal exercise test (CPET or ECG) and a single stage, high intensity, individualized ESE assessment, into a true combined evaluation (CPET–hiESE). This provides a feasible, high quality and time-efficient modification to current CPET–ESE protocols, based on individualized physiological data, which could have potential advantages in translating into heterogeneous clinical populations. Importantly, it provides the same accuracy in quantifying cardiac reserve using speckle tracking and TDI to current protocols, but at lower work rates and perceived exertion. This protocol could provide a basis for more uniform practice in combined multimodal cardiorespiratory functional assessment, opening more avenues for its use in longitudinal follow-up, risk stratification, and clinical research.

## Supplementary Material

qyae021_Supplementary_Data

## Data Availability

Consent for public sharing of research data was not given by participants for this study. Data are available to bona fide researchers subject to a data access agreement upon contacting the corresponding author.

## Lead author biography

Craig A. Williams is Professor of Paediatric Physiology and Health and Director of the Children’s Health and Exercise Research Centre (CHERC) based in the Exeter Medical School at the University of Exeter. He is the Editor-in-Chief of the journal Pediatric Exercise Science and for the last 30 years has specialized in the cardiovascular and metabolic responses to exercise of young people, healthy and those with complex medical conditions. His laboratory studies utilize *in vivo* techniques in exercise echocardiography and cardiopulmonary testing, near-infrared spectroscopy, and MRI, while translational work has led to clinical exercise intervention trials for adolescents with congenital heart disease.
