# Supplementary material for: A new protocol for a single-stage combined cardiopulmonary and echocardiography exercise test: a pilot study
Source: Eur Heart J Imaging Methods Pract. 2024 Mar 21;2(1):qyae021. doi: 10.1093/ehjimp/qyae021 (PMC11195695; doi:10.1093/ehjimp/qyae021)
Supplement: qyae021_Supplementary_Data [file qyae021_Supplementary_Data.docx]

**Supplementary Materials**

**Dorobantu et al:** A new protocol for a single stage combined cardiopulmonary and echocardiography exercise test: a pilot study

*Left ventricular ejection fraction (LVEF) and right ventricular fractional area change data (RV-FAC)*

LVEF and RV-FAC were secondary measurements for this study, as previous experience with high intensity stress echocardiography in our centre showed that both volume and area measurements are less reliable from stage to stage, due to geometrical variations in views and breathing movement artefacts, to a higher degree than semiautomated measurements such as speckle tracking. LVEF was measured using the Simpson Biplane method from apical four chamber and apical two chamber standard views, when both were of sufficient quality for adequate endocardial border tracing. RV-FAC was calculated from end diastolic and end systolic RV areas, as a percentage of change. When speckle tracking was not adequate in the main analysis, LVEF and RV-FAC were not attempted, due to poor endocardial border definition.

| **Supplementary Table 1.** Agreement between smESE , hiESE-GET and hiESE-HR testing modalities | | | | | | |
| --- | --- | --- | --- | --- | --- | --- |
|  | LoA 95% CI | Mean difference | outside LoA, n (%) | Concordance correlation | Difference-Mean correlation | Bradley Blackwood p value |
| **smESE vs hiESE-GET** | | | | | | |
| LVEF | [-8;8.6] | 0.3 | 1 (8.3) | 0.11 | 0.35 | 0.5 |
| RV-FAC | [-9;5.5] | -1.74 | 0 (0) | 0.66 | -0.17 | 0.9 |
| **smESE vs hiESE-HR** | | | | | | |
| LVEF | [-7.1;9.8] | 1.42 | 1 (7.7) | 0.35 | -0.09 | 0.7 |
| RV-FAC | [-8.6;8] | -0.3 | 0 (0) | 0.67 | -0.72 | 0.15 |
| **hiESE-GET vs hiESE-HR** | | | | | | |
| LVEF | [-7.7;8.5] | 0.41 | 0 (0) | 0.49 | 0.02 | 0.07 |
| RV-FAC | [-15.2;12.5] | -1.32 | 1 (7.7) | 0.31 | 0.06 | 0.8 |
| CI, 95% confidence interval; hiESE-GET, high intensity exercise stress echocardiography based on gas exchange threshold; hiESE-HR, high intensity exercise stress echocardiography based on peak heart rate; LoA, limits of agreement; LVEF, left ventricle ejection fraction; RV-FAC, right ventricle fractional area change; smESE, standard maximal exercise stress echocardiography. | | | | | | |

**Supplementary Figure 1.** Bland-Altman plots showing agreement in left ventricular ejection fraction (LVEF) and right ventricular fractional area change (RV-FAC) between smESE and hiESE-GET (**A.** LVEF **B.** RV-FAC), between smESE and hiESE-HR (**C.** LVEF **D.** RV-FAC) and between hiESE-GET and hiESE-HR (**E.** LVEF **F.** RV-FAC). Black horizontal line shown null mean difference. Horizonal green dashed line shows actual mean difference. Light blue area shows 95% limits of agreement (LoA). Red dots show data pairs. Agreement data in Supplementary Table 1.

**
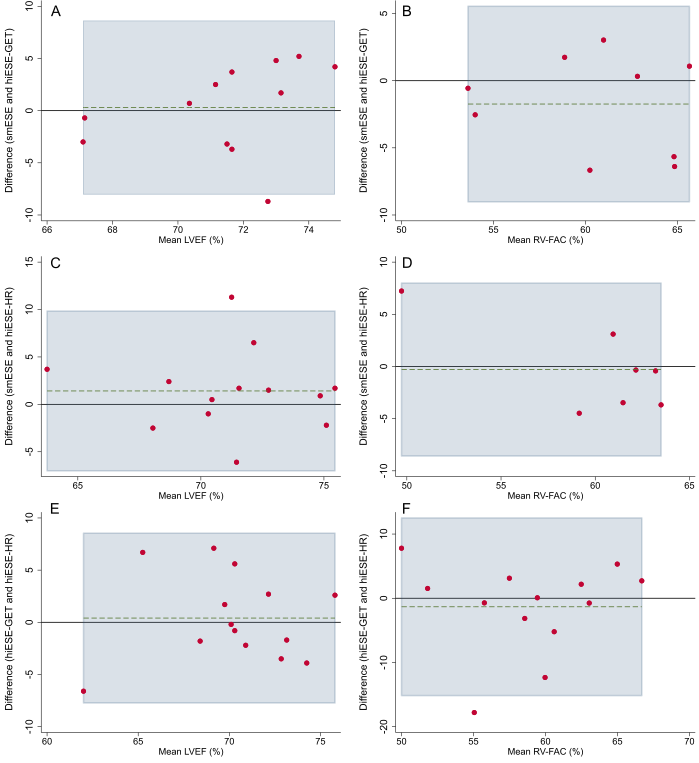
**
